# Are We Paving the Way to Dig Out of the “Pandemic Hole”? A Narrative Review on SARS-CoV-2 Vaccination: From Animal Models to Human Immunization

**DOI:** 10.3390/medsci9030053

**Published:** 2021-07-30

**Authors:** Giuseppe Tardiolo, Pina Brianti, Daniela Sapienza, Pia dell’Utri, Viviane Di Dio, Giuseppe Rao, Rocco Salvatore Calabrò

**Affiliations:** 1 Department of Veterinary Sciences, University of Messina, Via Palatucci snc, 98168 Messina, Italy; gtardiolo@unime.it; 2 Unit of Dermatology, IRCCS San Raffaele Hospital, Via Olgettina 60, 20132 Milan, Italy; brianti.pina@hsr.it; 3 Department of Biomedical and Dental Sciences and Morphofunctional Imaging, University of Messina, Piazza Pugliatti 1, 98122 Messina, Italy; daniela.sapienza@unime.it; 4 IRCCS Centro Neurolesi “Bonino Pulejo”, Via Provinciale Palermo, Contrada Casazza, 98124 Messina, Italy; pia.dellutri@irccsme.it (P.d.); viviane.didio@irccsme.it (V.D.D.); giuseppe.rao@irccsme.it (G.R.)

**Keywords:** animal models, COVID-19, SARS-CoV-2, vaccines, human immunization

## Abstract

The Severe Acute Respiratory Syndrome Coronavirus 2 (SARS-CoV-2) is a new pathogen agent causing the coronavirus infectious disease (COVID-19). This novel virus originated the most challenging pandemic in this century, causing economic and social upheaval internationally. The extreme infectiousness and high mortality rates incentivized the development of vaccines to control this pandemic to prevent further morbidity and mortality. This international scenario led academic scientists, industries, and governments to work and collaborate strongly to make a portfolio of vaccines available at an unprecedented pace. Indeed, the robust collaboration between public systems and private companies led to resolutive actions for accelerating therapeutic interventions and vaccines mechanism. These strategies contributed to rapidly identifying safe and effective vaccines as quickly and efficiently as possible. Preclinical research employed animal models to develop vaccines that induce protective and long-lived immune responses. A spectrum of vaccines is worldwide under investigation in various preclinical and clinical studies to develop both individual protection and safe development of population-level herd immunity. Companies employed and developed different technological approaches for vaccines production, including inactivated vaccines, live-attenuated, non-replicating viral vector vaccines, as well as acid nucleic-based vaccines. In this view, the present narrative review provides an overview of current vaccination strategies, taking into account both preclinical studies and clinical trials in humans. Furthermore, to better understand immunization, animal models on SARS-CoV-2 pathogenesis are also briefly discussed.

## 1. Introduction

The year 2020 is known as the year in which the first pandemic of the globalized era affected the world. The new coronavirus infectious disease, termed as COVID-19, is threatening the worldwide population, causing highly infectious pneumonia [1,2]. This disease appeared for the first time ever in Wuhan (Hubei, China) in December 2019, and, currently, its spreading occurs in every country [1,2]. The disease is the consequence of the Severe Acute Respiratory Syndrome Coronavirus 2 (SARS-CoV-2), a new virus belonging to the family of taxonomic *Coronaviridae* [3]. These viruses contain positive-sense single-stranded RNA and are known for the potential of infecting several animal species. The consequent illness presents various symptoms that are similar to the common cold or severe respiratory syndrome [1]. The Coronaviruses (CoVs) include a wide family of viruses, with the following classification genera: α, β, γ, and δ [3,4,5]. The SARS-CoV-2 was classified as β-coronavirus, showing nucleic acids sequence similarity with the previous SARS-CoV and the Middle East Respiratory Syndrome Coronavirus (MERS-CoV). An investigation by electron microscopy revealed a surface showing a crown-like morphology because of the spike glycoproteins presence [6]. These glycoproteins are composed of an ectodomain with two units: a receptor-binding unit called S1 and a membrane-fusion unit called S2. Therefore, to infect host cells, the S1 unit establishes a bind with a cell surface receptor by a receptor-binding domain (RBD), whereas the S2 unit mediates the fusion of the host cellular and viral membranes. In this manner, the nucleic acid of the virus can penetrate the host cells [7]. The Angiotensin-Converting Enzyme 2 (ACE2) is a functional receptor involved in the infection process [8], and its expression was observed in various animal species that can be potential SARS-CoV-2 natural hosts (e.g., fish, amphibians, birds, reptiles, and mammals). This receptor is highly expressed in the lung, intestine, testis, and kidney [6]. Studies conducted on human colon epithelial, lung, and patients’ endothelial glomerular capillary loops highlighted morphological and/or proteomic proofs of SARS-CoV-2 infection and host-viral protein interaction [9,10]. The cells of human autoptic samples from the respiratory tract presented the highest levels of SARS-CoV-2 RNA copies compared to lower levels observed in kidney, liver, heart, brain, and blood cells, thus showing a broad organotropism [11].

Wang et al. [12] were the first that depicted the main features of this new infectious syndrome. Pathogenetically, the infection process starts when the virus passes the nasal and larynx mucosa to enter the respiratory tract and then reach the lungs [13]. Then, the virus causes viremia by entering the peripheral blood, thus targeting organs that express the ACE2 receptor, including the heart and renal and gastrointestinal tract [13]. The presence of the virus in the gastrointestinal tract explains why it was also found in the feces samples [12,14]. The onset of the respiratory syndrome appears about eight days after the infection, with early symptoms reported, including fever and cough, leading to an aggravation until 14 days from the onset [12,13]. Initially, the blood cell counts in peripheral blood appear normal or slightly low, showing eventual lymphopenia [12] that can affect antibody production. If the immune system is effective throughout the acute pneumonia phase, the virus is suppressed, and recovery occurs. On the contrary, if the patient is in advanced age, the clinical picture may become severe. Furthermore, the non-survivors showed higher neutrophils counts, D-dimer levels, blood urea nitrogen, creatinine, and inflammatory cytokines compared to survivors [13].

### 1.1. Epidemiological and Clinical Features

In the past, CoVs were known for causing mild respiratory and gastrointestinal disease [15]. After the SARS outbreak of 2002, CoVs showed to have the capacity for epidemic spread and significant pathogenicity in humans. In recent two decades, the three new β-coronaviruses (namely, SARS-CoV, MERS-CoV, and SARS-CoV-2) had their spillover event, crossing the species barrier. Since then, these viruses have provoked significant human outbreaks characterized by high case-fatality rates [16,17,18]. To date, the SARS-CoV-2 is the newer entry to human pathogenic CoVs (hCoVs). Although hCoVs showed a comparatively low overall pathogenicity potential, such viruses can provoke severe respiratory or sepsis-like illness in immunocompromised people, infants, older people, and subjects with pre-existing pulmonary disorders [19,20]. By contrast, the novel CoVs may cause severe clinical pictures, with morbidity and case-fatality ratios higher than those by hCoVs. Indeed, the COVID-19 disease presents some symptoms, such as cough and fever, which in 8–19% of patients subacutely evolve to respiratory distress and acute respiratory distress syndrome. Old people and those with underlying comorbidities such as cardiovascular disease, diabetes mellitus, chronic pulmonary disorders, or renal disease are especially at risk of developing the disease [12,14,21,22]. Estimates concerning patients affected by COVID-19 who developed respiratory symptoms requiring supplemental oxygen was approximately 14%, and about 5% develop a need for mechanical ventilation [14,22,23]. SARS-CoV-2 causes a pulmonary pathology that shows diffuse alveolar damage and focal reactive hyperplasia of pneumocytes with patchy inflammatory cellular infiltration and intravascular thrombosis [24,25]. The severe pulmonary inflammatory infiltrates pulmonary tissue and impedes alveolar gas exchange. Besides, one fifth of hospitalized patients develop significant cardiovascular morbidity, characterized by troponin rise, tachyarrhythmias, and thromboembolic events, which are strongly associated with mortality risk [26,27,28]. Intensive care level supports are required by SARS-CoV-2 patients that manifest severe pneumonia with hypoxic respiratory failure of subacute onset evolving into acute respiratory distress syndrome. The clinical frame shows fevers, lymphopenia, highly elevated C-reactive protein, proinflammatory cytokines, serum ferritin, and D-Dimers leading to vasculitis and hypercoagulability [29,30].

### 1.2. Pharmacological Treatments

To date, a specific treatment for COVID-19 is not available yet. Management of SARS-COV-2 infection is directed firstly to manage/improve the clinical manifestations and to provide supportive care, as severe respiratory failure is the most common cause of death [31]. At present, there are many potential drugs for COVID-19 that affect virus binding or enzymatic activities involved in viral replication and transcription [31]. The safety and efficacy of several molecules, including RNA synthesis inhibitors, remdesivir, neuraminidase inhibitors, peptides, anti-inflammatory drugs, and HIV protease inhibitors, like lopinavir, have been confirmed by different clinical trials [32]. In particular, remdesivir received the authorization in around 50 countries for emergency use to treat COVID-19, considering its efficacy in treating previous CoV epidemics [33]. Unfortunately, the WHO in November 2020 included a conditional recommendation against the use of remdesivir for the treatment of COVID-19 for the high mortality despite the use of it. Current strategies are evaluating remdesivir in combination with modifiers of the immune response (e.g., the Janus kinase [JAK] inhibitor baricitinib in ACTT-2 and interferon beta-1a in ACTT-3) [31].

Immunomodulators and cytokine antagonists are also considered potential effective strategies. In this regard, tocilizumab, a recombinant humanized monoclonal antibody (mAb) raised to the IL-6 receptor, is believed to timely control the cytokine storm at the early stage in order to improve the success rate of treatment and reduce the mortality rate [34]. Regarding the antiviral activity of Type 1 interferons (IFN-1), their beneficial effects are at an early stage of infection, as, at a later stage, they may worsen the cytokine storm and exacerbate inflammation [32].

Chloroquine and hydroxychloroquine have also been used due to their immunomodulatory, rather than suppressive, characteristics. Because infection, inflammation, and other factors may lead to disseminated intravascular coagulation, respiratory distress, and multiorgan failure with a worse prognosis [32], the utility of combined administration intravenous immunoglobulin G and low molecular weight heparin anticoagulant therapy was used with positive results for the direct neutralization of the virus, controlling a cytokine storm, Th1/Th17, and the complement cascade activation and immunomodulating the hypercoagulable state. No severe side effects were reported. No specific virus was detected in CP before transfusion; indeed, plasma was subjected to methylene blue photochemistry to inactivate residual viral particles. Patients did not develop transfusion-related lung damage.

Among the passive immunotherapies based on neutralizing antibodies, randomized controlled trials of COVID-19 convalescent plasma as well as anti-SARS-CoV-2 spike protein mAbs have been found potentially effective in the early disease course (i.e., less than 72 h from symptoms). Nonetheless, the hyperimmune *serum* has several advantages over convalescent plasma (e.g., a smaller reinfusion volume, an easier administration route, and easier preservation) and mAbs (more diversified neutralizing antibodies (nAbs) against emerging variants of concern, and far cheaper) [35]. The development, production, standardization, assessment, and commercialization of new specific drugs to treat this novel virus currently require long-term experimentation; therefore, at present, the best way to deal with a pandemic is immunization using the currently available vaccines.

In this global frame, the present narrative review aims at investigating the current vaccination strategies, taking into account both preclinical studies and clinical trials in humans. For a better understanding of the immunization process, animal models on SARS-CoV-2 pathogenesis are also briefly discussed.

## 2. Search Strategy

Preclinical and clinical studies were identified by searching on Scopus, PubMed, Web of Science, and Cochrane database. All the studies fulfilling our selected criteria and published between January 2020 and May 2021 were evaluated for possible inclusion. The search combined the terms “SARS-CoV-2” and/or “animal models” and/or “vaccination” and/or “COVID-19” and/or “immunization” and/or “vaccines” and/or “humans”. Only texts in English were selected, and duplicates were removed. All articles have been evaluated according to the title, abstracts, and text. Studies with other viruses or vaccines than SARS-CoV-2 were not considered.

## 3. Towards Understanding the Pathogenic Mechanisms of SARS-CoV-2: Insights from Animal Models

Nowadays, in the globalized era, viral diseases can represent severe global public health threats, as shown by the SARS-CoV-2 pandemic [31].

An increased understanding concerning the pathogenesis and mechanisms of transmission can be achieved by the employment of some animal models that recapitulate the disease’s clinical features [15]. However, we report some relevant studies that employed animal models to investigate the SARS-CoV-2 pathogenesis, which is fundamental to guide toward vaccine development [15], although this issue has not been properly addressed by the media, generating misinformation and fake news.

Among the animal models, laboratory mice are undoubtedly the most used [15]. Notwithstanding, mice showed to be less susceptible to the SARS-CoV-2 infection, and this can be correlated to mouse ACE2 receptor incompatibility with S glycoprotein. Different approaches have been used to overcome the incompatibility issue, as a mouse-adapted SARS-CoV-2 strain and human ACE2-transgenic mice, including C57BL/6 and BALB/c mice. Even though exposed to the infection, mice manifested transient infection and viral replication that is limited by the induction of innate immune response [36]. Nonetheless, in infected human ACE2-transgenic mice, SARS-CoV-2 showed efficient replication in the lower respiratory tract, albeit it exclusively caused a mild or moderate interstitial pneumonia with no severe clinical symptoms [37]. Notwithstanding that an active viral replication was observed in their lungs, 40% of mortality occurred by five days of post-infection because of brain tropism [38]. On the contrary, early active virus replication in the lungs of the SARS-CoV-2 hamster model was observed, as they manifested an important lung pathology that showed necrotizing bronchiolitis, massive leukocyte infiltration, and edema [36].

Ferrets have been employed to investigate respiratory diseases and the transmission mechanisms of several respiratory viruses, including influenza and respiratory syncytial virus [39]. Ferrets are considered smart models of study due to their similarities with humans in tissue architecture and anatomy of the respiratory system concerning the receptor distribution, submucosal gland density in the bronchial wall, and the number of terminal bronchioles [39]. In infected or contact-exposed ferrets (3–4-month-old), efficient viral replication of SARS-CoV-2 in the upper respiratory tract was also observed, including nasal turbinate, soft palate, and tonsils. As in humans, infected/contact-exposed ferrets also showed efficient viral shedding in their biological fluids and feces [40].

Cats were also employed as an animal model, showing an age-related difference in viral replication, with juvenile cats being more prone to viral replication [40]. Among other animal models infected with SARS-CoV-2, dogs showed a minor susceptibility. On the contrary, no susceptibility was observed in livestock, including pigs, chickens, and ducks [40]. The potential for reservoir and successive transmission from companion animals is currently unknown and deserves more studies [41].

Concerning non-human primate models, macaques infected with SARS-CoV-2 showed a productive infection with clinical signs ranging from asymptomatic in *cynomolgus macaques* to moderate disease in *rhesus macaques* [42,43,44]. Non-human primate models can contribute to the assessment and release of preventive and therapeutic strategies, including vaccination, although these models fail to recapitulate the entire severe disease and critical conditions observed in COVID-19 patients [42,43,44].

## 4. Preclinical Research for SARS-CoV-2 Vaccine Development: An Overview

At present, vaccines represent the most effective strategy for controlling and preventing the disease from spreading. The rapid rates of infectivity showed by SARS-CoV-2 needed an urgent response worldwide by the scientific community to speed up the development of vaccines to counteract the infection spreading. To this aim, different vaccines formulations have been used, including inactivated, non-replicating viral vector, replicating viral vector, protein subunit, RNA and DNA vaccines, and virus-like particle vaccines. Among them, mRNA and non-replicating viral vector vaccines have recently been displayed to be the most promising preparations, and, therefore, these are currently used in human immunization [45]. Table 1 reports the non-human primate animal model employed for the development of leading COVID-19 vaccines.

The most common form used for vaccine formulation is the *inactivated vaccine,* in which the whole virus is used. The virus can be inactivated either physically (by heat) or chemically (e.g., β-propiolactone). Their production usually occurs using the *Vero* cells lines, in which the culture supernatant is purified and formulated with or without adjuvant [46,47,48]. The production of this type of vaccine is easy, even though it requires a biosafety level 3 facility. Companies that currently are developing *inactivated vaccines* are: the Sinovac Biotech, Ltd. (Beijing, China); the Wuhan Institute of Biological Products (China); the Beijing Institute of Biological Products (Beijing, China); the Chinese Academy of Medical Sciences; the Research Institute for Biological Safety Problems (Zhambyl, Kazakhstan Kazakhstan); Bharat Biotech (India); Shenzhen Kangtai Biological Products Co., Ltd. (Shenzhen, China); Valneva, National Institute for Health Research (London, UK); and Erciyes University (Kayseri, Turkey) [49].

In China, the company Sinovac Biotech developed the PiCoVacc, a β-propiolactone *inactivated vaccine* (now known as CoronaVac). This preparation was displayed to induce the production of nAbs in an animal model that used *rhesus macaques.* To obtain this result, animals received, at one-week intervals, the administration of three intramuscular (IM) injections (3 or 6 µg PiCoVacc adjuvanted with alum hydroxide each) [46]. After the second boost before the virus challenge test, the nAbs titers rose to about 50. These levels are similar to those observed in the *serum* from the COVID-19 recovered subjects [15]. Compared to the control, immunized monkeys receiving a 3 µg/dose displayed a partial protection response after a direct intratracheal administration of the virus into the lungs, with viral loads detected in the pharynx, anal canal, and pulmonary tissues [15].

The *inactivated vaccine* BBIBP-CorV was developed by the Beijing Institute of Biological Products. Preclinical studies were performed to assess its effect in *cynomolgus macaques* using two-dose immunizations regimens [15]. At both doses, respectively low (2 µg/dose) and high (8 µg/dose), the formulation provided efficient protection. Before the intratracheal challenge with 10^6^ TCID_50_ of the virus, the mean titers of the nAbs in the low-dose and high-dose groups reached 215 and 256, respectively. In the lungs of immunized animals of both groups, no viral load was detected seven days post-infection. Notably, no antibody-dependent enhancement of infection was noticed among the vaccinated animals [15].

*Live-attenuated vaccines* contain live, whole bacterial cells or viral particles. The microorganisms are submitted to treatments to reduce virulence, although they still retain some antigenicity after their attenuation [50]. The reduction of virulence can be obtained by artificial mutations, gene deletions, or by selection from nature. These vaccines may simulate naturally occurring recessive infections and induce comprehensive, stable, and persistent responses. The administration can be performed via different routes, including oral, nasal, and/or aerosol. Moreover, these vaccines can induce antibody, cell, and mucosal immune responses [45,50]. At present, only one live-attenuated vaccine entered a clinical trial (NCT04619628), which is co-developed by Codagenix Inc. (Farmingdale, NY, USA) in collaboration with the Serum Institute of India.

A further typology of vaccines is the *non-replicating viral vectors*, thanks to the growing contribution of genetic engineering technology. These viral vectors can transport a foreign gene encoding a polypeptide, antigen, or other molecules transported to the host cell [45,50,51]. Two types of viral vectors, those non-replicating and those replicating, are known nowadays. The vaccines formulations that use non-replicating viral vectors are deficient in those functions essential for viral replication. Several types of non-replicating viral vectors are available: *Poxvirus*, *Adenovirus*, *Alphavirus*, *Herpes simplex* virus, *Measles* virus, and other viral vectors. The Adeno-associated viruses are probably those widely investigated and employed for vaccine development due to their safety, ease of production, and capacity to be delivered to numerous host cells through various routes [51].

Currently, eleven non-replicating viral vectors vaccines are available and under clinical assessment. Among them, eight use Adeno-associated virus and one a modified vaccinia virus Ankara [49].

The replication-deficient chimpanzee viral vector is another vaccine based on the Adeno-associated virus vector, incorporating a weakened version of the adenovirus containing the gene encoding wild-type S-protein (ChAdOx1nCoV-19) [52]. The University of Oxford (Oxford, UK), in collaboration with the company AstraZeneca (Cambridge, UK), developed this preparation. The preclinical tests involved *rhesus macaques*, in which the viral load in the lung was significantly reduced after the challenge test administration with 2.6 × 10^6^ TCID_50_ live SARS-CoV-2 (combined intratracheal, intranasal, oral, and ocular). No pulmonary pathology in the vaccinated group was observed, and, interestingly, low T cell responses were detected.

The collaboration between Janssen Pharmaceutical Companies and Harvard and MIT led to the development of a non-replicating adenovirus vector vaccine candidate based on Ad26. This vaccine is currently commercialized by *Johnson & Johnson* and used for human immunization [53]. *Rhesus macaques* were for preclinical investigation used, receiving the vaccine intramuscularly with a single dose of the d26-vectored vaccine encoding for seven versions of the S-variant or the sham control and then were performed challenge tests with SARS-CoV-2 [53]. The optimal Ad26 vaccine, Ad26.COV2.S or proline stabilizing mutations (S.PP), induced robust nAb responses with median titers of 113 (range 53–233). Interestingly, these titers were 4-fold higher than those from human convalescent sera [53]. The high nAb titers induced by the vaccine administration can be considered a potential biomarker and then correlated with its protective efficacy.

Engineered viruses or bacteria are used in *replicating vector vaccines* technology for the vaccine vector to express a target gene in the host cell. Furthermore, viruses that do not replicate efficiently or those that cause no disease in humans can also be used [45,54]. Replicating vector-based approaches, using, e.g., measles virus, influenza virus, and vesicular stomatitis virus, can trigger strong immune responses because the vector will propagate in vivo to some extent. Currently, only a few phase I clinical trials are investigating the replication of active vectors against SARS-CoV-2.

Another technology for vaccine development is based on *recombinant protein vaccines.* These preparations are composed of recombinant subunit proteins, peptides, or virus-like particles (VLPs). These can be obtained using several systems based on, e.g., E. coli, yeasts, plants, insect cells, and mammalian cells [50,54]. The primary target antigens chosen for this purpose are usually the RBD as S1, S-protein, or N-protein. The preparation of these vaccines includes the combination of potent adjuvants to improve immunogenicity and efficacy. Differently from other vaccine technologies, recombinant protein vaccines are safe and easily manufactured by recombinant molecular techniques [54].

In the USA, a recombinant protein vaccine was developed by the company Novavax. This vaccine uses the full-length S-protein stabilized in its prefusion form, and it is produced in insect cells [55,56]. In a preclinical investigation, *cynomolgus macaques* received the administration of two doses of 2.5, 5, or 25 μg NXV-CoV2373 at a 21-day interval [55], with positive results. Notably, only one monkey (1/4) in the middle-dose (5 μg) group showed detectable sgRNA levels in the fluid of bronchoalveolar lavage. Moreover, seven days post-challenge test, only little to no inflammation was noticed in animals’ lungs [55,57].

The results of preclinical investigations on *rhesus macaques* were published for another three vaccines: (i) an RBD-dimer vaccine developed by the Zhifei Longcom Biopharmaceuticals and the Institute of Microbiology (Chinese Academy of Sciences); (ii) an RBD monomer developed by the West China Hospital at Sichuan University; and (iii) a native-like S-trimer (wild-type) vaccine based on Trimer-Tag technology developed by Clover Biopharmaceuticals (China) in collaboration with GSK (London, UK) and Dynavax (Emeryvill, CA, USA).

An important form of exogenous expression of the viral capsid protein is represented by the *virus-like particle* *vaccines*. The morphological structure is highly similar to that of natural viruses, with considerable immunogenicity and better safety [58]. Compared with traditional attenuated or inactivated vaccines, highly purified VLP vaccines show important advantages: they comprise a single component, have no viral nucleic acid, have a good safety profile, and offer high immunogenicity. Nevertheless, a need for a suitable scale-up preparation, assembly, and formulation process may hinder their development.

In clinical trials, there are two VLP-based COVID-19 vaccines under investigation. The first one is developed by SpyBiotech Ltd. (UK) and the Serum Institute of India in phase I/II (ACTRN12620000817943), named RBD-HBsAg VLPs, whereas the other one was developed by Medicago Inc. (Quebec city, CA, USA) (NCT04636697), and it is a plant-derived VLP adjuvanted with GSK or Dynavax adjuvant.

Another technology of vaccine is the *DNA vaccines*. They are composed of a plasmid containing various regulatory elements, which contribute to ensuring efficient production of the plasmid in bacterial systems, including an origin of replication, a selectable marker, and an expression cassette containing the gene of interest under a eukaryotic promoter [50,54]. The production of these vaccines is simple and easy, and it is based on well-established fermentation technologies in *E. coli*. These vaccines can induce humoral and cellular immune responses in systemic and mucosal compartments [45].

However, DNA vaccines have a disadvantage consisting of the poor efficiency of transfection and the need for delivery devices. Therefore, this may limit their future application [45]. In Korea, the Genexine Consortium reported preclinical data for the GX-19 DNA vaccine. This vaccine is composed of the whole ectodomain of the S gene and the N-terminal tissue plasminogen activator signal sequence [59]. In the preclinical investigation, *cynomolgus macaques* were employed. At approximately 3-week intervals, three macaques were vaccinated three times using the GX-19. The vaccination induced an S-specific, Th1-biased immune response. Moreover, several DNA vaccine candidates expressing different forms of the SARS-CoV-2 S-protein were reported by the Harvard Medical School [60,61]. The preclinical experimentation evaluated the vaccine candidates on 35 *rhesus macaques* with a regimen of three IM injections. After the vaccination, the treated macaques developed humoral and cellular immune responses, including nAb titers (median 74), comparable with those encountered in the *serum* of convalescent humans and macaques infected with SARS-CoV-2.

Finally, *RNA vaccines* represent an advanced and promising vaccine technology. Currently, RNA vaccines are worldwide used for human immunization. The advanced technology used for their production is based on characteristics of both *subunit vaccines* and *live-attenuated vaccines*. These vaccines can induce both humoral and cellular immune responses, and their production is fast and flexible. RNA vaccines include messenger RNA (mRNA) and self-amplifying RNA (Replicon) vaccines [50,61]. The RNA vaccines production is based on the use of lipid nanoparticles technology, which can improve their administration intramuscularly or intradermic [45].

Differently from DNA vaccines, the RNA ones should not integrate into the genome of the immunized host and directly express the antigen in the cytoplasm. In light of this, RNA vaccines might be more beneficial in stimulating antigen-specific immunity [50]. However, the genome integration possibility of RNAvaccines needs long-term monitoring in vaccinated populations to fully justify this issue.

The company Moderna (USA) developed the mRNA-1273 vaccine, which contains the prefusion-stabilized S-protein with two proline substitutions, a native furin cleavage site and a transmembrane-anchored protein [62]. The vaccine was synthesized in vitro and encapsulated into lipid nanoparticles, and it was evaluated in the preclinical investigation using *rhesus macaques*. The animals received an IM injection of 10 or 100 µg mRNA-1273 in a prime-boost regimen with a 4-week interval [63]. In the low- and high-dose groups, the GMTs of the nAbs reached 501 and 3481, respectively, after the boost. Notably, the vaccine also induced a good CD4 and an increase in T-cell responses. As no viral replication was detectable in nasal swabs for the high-dose group and limited inflammation cytokine induction was noticed in the lungs of animals in both vaccine groups, it was suggested that this vaccine could offer rapid and potentially enduring protection.

The collaboration established between the company BioNTech (Germany) with Fosun Pharma (China) and Pfizer (USA) led to the development and production of another mRNA-based vaccine. The preclinical investigation was conducted for both BNT162b1 and BNT162b2, two vaccine candidates that contain nucleoside-modified messenger RNA encoding for immunogens derived from the spike glycoprotein (S) of SARS-CoV-2. Interestingly, lipid nanoparticles are employed for the final formulations [50,64]. In mice, these vaccines induced a dose-dependent antibody response with high virus-entry inhibition titres and strong T-helper-1 CD4^+^ and IFNγ^+^CD8^+^ T cell responses. Rhesus macaques), instead, received prime-boost vaccination with BNT162b that induced high SARS-CoV-2-neutralizing geometric mean titres. Vaccinated macaques showed protection against challenge with SARS-CoV-2. Specifically, BNT162b2 protected the lower respiratory tract against the presence of viral RNA and showed no evidence of disease exacerbation.

Moreover, the company Walvax Biotechnology (China), in collaboration with the Academy of Military Medical Sciences (China) and Suzhou Abogen Biosciences Co., Ltd. (China), developed another mRNA vaccine candidate, encapsulated in liquid nanoparticles which encoded the RBD and named ARCoV. The preclinical investigation involved mice and a non-human primate model employing cynomolgus monkeys with promising results [65]. ARCoV is also under investigation in phase I clinical trials.

## 5. Vaccine Development/Marketing in Humans: A Brief Overview

Many types of vaccines used in animal models have also been tested in humans with positive results and then approved by the national regulatory agencies (see Table 2).

A new platform for vaccine production is represented by the mRNA vaccines, although their development requires further research, including clinical trials and post-marketing evaluation. Nonetheless, while the vaccines were developed quickly in response to the pandemic, they have been proven to be safe and effective not only in standard clinical trials but in real-world conditions. The vaccines in question use modified mRNA to provide instructions for cells to produce SARS-CoV-2 virus spike proteins that are able to trigger an immune response. Notably, the cells quickly break down the mRNA, which in any case may interfere with the host DNA, as instead claimed by some pandemic “negationists”. In fact, some have weaponized their novelty to spread misinformation and unfounded claims about their safety, including nucleic acid modifications.

In phase III trials, the two mRNA vaccines authorized, i.e., Pfizer/BioNTech and Moderna, had an efficacy of 94% or higher. Moreover, the Centers for Disease Control and Prevention study released in March found that, in real-world conditions, the two vaccines were 90% effective in preventing infections. In particular, the emergency use of mRNA-1273 (Moderna) was urgently authorized by the U.S. FDA to counteract COVID-19 infection in subjects 18 years old and older on 18 December 2020.

A randomized, observer-blinded, placebo-controlled phase III trial enrolled (30,420) volunteers receiving either the vaccine or placebo 28 days apart. Overall, the mRNA-1273 vaccine was 94.1% effective at preventing laboratory-confirmed COVID-19 onset, as well as severe disease, with higher rates of moderate but transient adverse events after vaccination [66].

Concerning the vaccine by Pfizer, two vaccine candidates, respectively BNT162b1 and BNT162b2, were assessed and compared in a randomized, placebo-controlled, observer-blinded, dose-escalation trial (NCT04368728) in both younger (aged 18–55 years) and older adults (aged 65–85 years). Although the antibody titers between the two vaccine types were similar, BNT162b2 was presented with fewer systemic side effects, thus configuring a more favorable safety profile. Then, following another pivotal efficacy trial [67], in which 43,548 people aged > 16 years received two doses of either placebo or BNT162b2, the vaccine was conditionally authorized for marketing by the European Commission [67].

Data from phase II/III clinical trials showed 79.34% efficacy of PiCoVacc, i.e., a β-propiolactone inactivated vaccine developed by Sinovac Biotech (Beijing, China) (NCT04560881, ChiCTR2000034780) with a good safety profile. This vaccine has been approved by the National Medical Products Administration and conditionally marketed on 31 December 2020, being the first native COVID-19 vaccine in China [48].

Other inactivated vaccines (such as the Sinopharm), as well as live-attenuated vaccines, have been authorized or are under investigation in China and India. Notably, the first-in-human trial against coronavirus was conducted by CanSino (Tianjin, China) using a non-replicating Ad5-based vaccine expressing the wild-type S-protein. Since then, other important trials have been conducted, leading to the authorization of important vaccines.

The European Medicines Agency (EMA) has conditionally authorized for marketing the vaccine AstraZeneca to prevent COVID-19 in people aged >18 years on 29 January 2021. Combined results from four clinical trials in the United Kingdom, Brazil, and South Africa demonstrated that this vaccine was safe and effective to prevent the disease. These studies involved around 24,000 people altogether, who received either the vaccine or were given a control injection (i.e., a dummy injection or another non-COVID vaccine), with an overall efficacy of about 70% [68]. As most of the participants in these studies were between 18 and 55 years old, there were not enough results in older participants to provide information for how well the vaccine will work in this group. However, after secondary analyses, the vaccine was considered safe also in people aged >60 and <80.

Data from a clinical trial involving people from different countries (i.e., USA, South Africa, and Latin America) showed that the vaccine Janssen was effective at preventing COVID-19 in 44,000 people aged >18 years. The trial (NCT04505722) found a 67% reduction in the number of symptomatic cases after 2 weeks from the vaccine administration (116 cases out of 19,630 people) compared with people who received the placebo (348 of 19,691 people). The side effects following vaccination were generally mild to moderate (i.e., pain at the injection site, headache, tiredness, muscle pain, and nausea) and cleared within a couple of days.

A novel DNA vaccine for SARS-CoV-2 called INO-4800 developed by Inovio Pharmaceuticals engineered a synthetic DNA vaccine targeting the MERS coronavirus spike (S) protein, the major surface antigen of coronaviruses and results in robust expression of the S protein in vitro. By using double-stranded DNA plasmids, it allows researchers to synthesize and code for the Spike protein, which will initiate an immune response. A Phase I, open-label trial in the United States (NCT04336410), is evaluating the safety, tolerability, and immunogenicity of INO-4800 on healthy volunteers between 18 and 50 years old. The vaccine will be injected intra-dermally, followed by electroporation [61]. The INO-4800 vaccine induces cellular and humoral host immune responses that can be observed within days following a single immunization, including antigen-specific T cell responses, functional antibodies which neutralize the SARS-CoV-2 infection by blocking the Spike protein binding to the ACE2 receptor, as well as the biodistribution of SARS-CoV-2 targeting antibodies to the lungs.

Finally, in Russia, the Gamaleya Research Institute worked on the development of a heterologous COVID-19 adeno-based vaccine that uses the Ad26 and Ad5 vectors carrying the gene for the full-length S protein (rAd26-S and rAd5-S). As a Russian phase III clinical found that the efficacy of this vaccine was 91.4% as per a point analysis performed 21 days after administering the first dose to volunteers (*n* = 22,714), this has been authorized with a procedure for emergency use [69].

Concerning recombinant vaccines, the NVX-CoV2373 (Novavax) in its Phase II/III trials presented absent or mild reactogenicity. Interestingly, the vaccine induced a strong Th1 response and a minimal Th2 response, and the addition of the adjuvant enhanced this immune response.

For the protection of the adolescent population, safe and effective vaccines are needed in order to facilitate in-person learning and socialization and contribute to herd immunity. A multinational, placebo-controlled trial involved 2260 adolescents (aged 12–15 years): 1131 received BNT162b2, and 1129 received placebo, confirming the safety and effectiveness of the vaccine. Indeed, Ab titers were high and side effects were mainly transient mild-to-moderate, including injection-site pain (up to 86%), fatigue (up to 66%), and headache (up to 65%) [70].

Another hot point concerns the efficacy of the current vaccines to fight virus variants. To this end, SAGE has reviewed all available data on the performance of the vaccines in the settings of the variants of concern. In clinical trials, the Jansen vaccine has been tested against a variety of SARS-CoV-2 virus variants, including B1.351 (first identified in South Africa) and P.2 (first identified in Brazil), and found to be effective. Good results have also been found for the Pfizer vaccine [71,72].

## 6. Towards Novel Vaccination Approaches: The Intranasal Route

Since SARS-CoV-2 primarily affects the respiratory tracts, including lungs, and then the generation of mucosal immunity is crucial for protection. Eliciting mucosal immunity would result in enhanced production of effective and specific IgA at the mucosal site, neutralizing IgG, as well as specific T-cell response. Indeed, the local mucosal immune response is important in this case to mitigate the replication of the SARS-CoV-2 virus in nasal epithelia. Owing to the route of infection and nature of SARS-CoV-2, the mucosal route of vaccine administration is being propagated as a more robust and viable route for COVID-19 vaccine delivery. A growing number of COVID-19 vaccine candidates are being developed and formulated to be administered intranasally. [73]

The most promising intranasal COVID-19 vaccine candidates are COVI-VAC from Codagenix (USA) and Serum Institute (India). This is a replicating viral vector-based RBD expressing vaccine developed by the University of Hong Kong and Beijing Wantai Biological Pharmacy (China). COVI-VAC (a single-dose intranasal live-attenuated vaccine) has proceeded to phase 1 trial [74], and it has been found to protect against all virus antigens/proteins and not just the spike protein, being potentially more beneficial.

The AdCOVID (developed by the collaborative work between Altimmune Inc. (USA) and the University of Alabama (USA)) is a replication-deficient human adenovirus 5 (hAd5) vectored single-dose intranasal vaccine encoding the receptor-binding domain of the spike protein of SARS-CoV-2, activating both mucosal and systemic immunity. It has been demonstrated that AdCOVID elicited strong serum neutralizing antibodies, T-cell response (CD4+ and CD8+ response with a Th-1 like a cytokine expression), and mucosal IgA in the respiratory tract. AdCOVID is reported to be stable over several months at room temperatures. The phase 1 clinical trial of AdCOVID is presently recruiting subjects [75].

Recently, the Washington University, School of Medicine, St. Louis (USA) is developing an intranasal non-replicating chimpanzee adenovirus vectored vaccine expressing the spike protein gene of SARS-CoV-2, and it has entered the phase of licensing agreement with Bharat Biotech India to manufacture the candidate (name BBV154). It has been shown that a single intranasal dose of the vaccine elicited significant neutralizing antibodies and T-cell response against the SARS-CoV-2 virus at 1-month follow-up after the day of immunization [76]. Finally, Meissa vaccine (San Francisco, CA, USA) has designed a candidate who is based on codon optimization technique, and the vaccine is currently in the preclinical GMP manufacturing stage using AttenuBlock™ proprietary technology. The company has used the same technology for the development of the respiratory syncytial virus intranasal vaccine candidate, which is currently in phase 2 trial [77].

## 7. Authors’ Point of View and Conclusions

The advent of the new infectious disease triggered by SARS-CoV-2, and the possibility to become an endemic virus, is opening the doors for the so-called “*age of pandemics*”. In this global frame, it is important to concentrate the efforts in clinical research to improve vaccination strategies and to provide immunization in countries in which a developed healthcare system lacks. For the protection of the majority of the world population, including high-risk groups, more than 200 COVID-19 vaccine candidates are under investigation by adapting previously developed vaccine platforms and applying knowledge of other infectious viruses, including HIV, influenza, and previous SARS-CoVs. Increased stability of the vaccine materials, improved pharmacokinetics and pharmacodynamics of the antigens, and enhanced immune response by co-delivered adjuvants are considered among the potential advantages of preparing COVID-19 vaccines with various nanotechnology platforms [78].

The virus mutations that frequently occur in the SARS-CoV-2 S protein represent a relevant challenge in the development and updating of COVID-19 vaccines. In fact, in the current efforts, this protein is the most common target antigen limiting the effectiveness of the first-generation vaccines against COVID-19 and even requiring the recovered patients to be vaccinated against new variants. Indeed, there have been growing descriptions of an increasingly prevalent SARS-CoV-2 variant phylogenetic cluster, which is associated with increased transmission in the UK (the same variant is now described in at least 25 other countries). Further variants of concern have been identified in South Africa, Brazil. and more recently in India, encompassing coding mutations from the wild-type sequence [79].

Therefore, an ideal COVID-19 vaccine platform must allow easy and fast adaptation of newly mutated and identified antigens. Ongoing clinical studies on COVID-19 vaccines are aimed at targeting the production of neutralizing antibodies against SARS-CoV-2, but the potential induction of memory T and B cells is fundamental to avoid, or at least counteract, the virus infection and spreading. In fact, effective and long-term protection from SARS-CoV-2 infection requires a well-orchestrated innate, humoral, and cellular immunity, which can be achieved by a vaccine integrating several potent antigens or antigen-encoding nucleic acids, co-delivering appropriate co-stimulatory molecules, and targeting certain immune cells. However, although vaccines are believed remarkably safe by the regulatory agencies (i.e., FDA, EMA, AIFA, etc.), also considering the few clinically significant post-approval adverse events, a more robust post marketing surveillance program is needed. Indeed, an association between the AstraZeneca vaccine (Vaxzevria/ChAdOx1 nCoV-19/AZD1222) and rare cases of thrombosis have been recently described in Europe. In fact, cases of thrombosis in unusual locations, including cerebral venous sinus, splanchnic vein, or pulmonary thrombosis, have been reported in the days after vaccination. These episodes were associated with low platelet levels and a strong increase in D-dimers with normal or low fibrinogen levels. For this reason, the vaccine has been permanently or temporarily suspended in different European countries, as far as middle April 2021. Later on, the FDA has suspended the administration of the *Johnson & Johnson*–Janssen vaccine after having identified eight cases of cerebral venous thrombosis with severe thrombocytopenia. These vaccines are still used in many countries, including Italy, but with limitations. Moreover, concerning vaccines using new technologies (i.e., mRNA ones), there is unmotivated fear of the potential long-term side effects due to mRNA/DNA interactions. Studies on safety that consider long-term follow up are needed to overcome this concern.

In conclusion, increased comprehension of the pathogenesis, transmission, and immune reactions against SARS-CoV-2 in animals and humans is required. Indeed, there are still gaps in our knowledge regarding the detection of the virus within the target population, the variability of the S protein, and the absence of standardized assays and/or proper animal models for COVID-19. However, several vaccine candidates have been developed and reached clinical trials, and other vaccine candidates are soon to be developed. In fact, as the COVID-19 pandemic is an ongoing, global concern, vaccines could be the only effective and economical means to manage this outbreak to dig out of the “pandemic hole”.

## Figures and Tables

**Table 1:** Overview of non-human primate animal models employed in the development of promising COVID-19 vaccines.

Company Developer	Vaccines Name	Vaccines Strategy	Non-Human Primate Model (Species)	Administration (Dose and Route)	Cost (Single Dose)
Sinovac	PiCoVacc (CoronaVac)	inactivated alum hydroxide	*Rhesus* *macaques*	3 or 6 µg (intramuscular)	10$
Beijing Institute of Biological Products	BBIBP-CorV	inactivated alum hydroxide	*Cynomolgus* *macaques*	3 or 6 µg (intramuscular)	NA
AstraZeneca	ChAdOx1nCoV-19	non-replicating viral vector	*Rhesus* *macaques*	2.5 × 10^10^ (intramuscular)	3.75$
Janssen (J&J)	Ad26.COV2.S	non-replicating viral vector	*Rhesus* *macaques*	10^11^ (intramuscular)	10$
Novavax	NVX-CoV2373	protein subunit	*Cynomolgus* *macaques*	2.5, 5, 25 µg (intramuscular, 0, 21)	16$
West China Hospital (Sichuan University)	RBD monomer	protein subunit	*Macaca* *mulatta*	20, 40 µg (intramuscular, 0, 7)	NA
Clover Biopharmaceuticals	S-trimer	protein subunit	*Rhesus* *macaques*	30 µg (intramuscular, 0, 21)	NA
Genexine Consortium	GX-19	DNA vaccine	*Cynomolgus* *macaques*	3 mg (electroporation, 0, 3, 5.5 weeks)	NA
Harvard Medical School Moderna	set of prototype vaccines expressing various forms of the spike (S) protein	DNA vaccine	*Rhesus* *macaques*	5 mg (intramuscular, 0, 3 weeks)	NA
	mRNA-1273	mRNA vaccine	*Rhesus* *macaques*	10, 100 µg (intramuscular, 0, 4 weeks)	32$
Pfizer	BNT162b2	mRNA vaccine	*Rhesus* *macaques*	30, 100 µg (intramuscular, 0, 21 days)	20$
Walvax Biotech	ARCoV	mRNA vaccine	*Cynomolgus* *macaques*	100, 1000 µg (intramuscular, 0, 14 days)	NA

**Table 2:** Summary of current progress in human vaccines development.

Vaccines Strategy	Company Developer	Protective Effects (Wild Type and Variants)
Inactivated virus (PiCoVacc)	Sinovac with National Institute for Communicable Disease Control and Prevention	- protective of effect of 67% in Chile - in vitro effectiveness against B.1.1.7 and B.1.351 spike-expressing recombinant virus
Inactivated Virus	Wuhan Institute of Biological Products, Sinopharm, with Wuhan Institute of Virology, Chinese Academy of Sciences	Very high direct protective effect (99.06%) -
Inactivated virus (BBIBP-CorV)	Beijing Institute of Biological Products, Sinopharm, with	- The protective effect was 79.34%
	Institute of Viral Disease Control and Prevention	- roughly equivalent against B.1.1.7 and B.1.351 variants
Virus vector (Ad5)	CanSino Biological Inc. with Beijing Institute of Biotechnology	protective efficacy against all symptoms of 68.83% (phase III clinical trial)
Virus vector (ChAdOx1)	University of Oxford with AstraZeneca	the protective effect was 76% (phase III clinical trial), a causal relationship between vaccine and thrombosis was found in rare cases (warning)
Liquid nanoparticle mRNA-1273	Moderna, with National Institute of Allergy and Infectious Diseases	- The protective effect was 94.1% - roughly equivalent against B.1.1.7 and B.1.351 spike-expressing virus
Liquid nanoparticle (BNT162b2)	BioNTech with Fosun Pharma and Pfizer	- the effective rate for the prevention of severe disease is 100% - roughly equivalent against B.1.1.7 and B.1.351 spike-expressing virus
Protein subunit (NVX-CoV2733)	Novavax	- 95.6% of protective effect (phase III trial in the UK)- - About 90% effective against the B.1.1.7 (UK) and B.1.351 (South Africa) variants
Virus-vectored (Ad26)	Janssen Pharmaceuticals Company	Effectiveness of about 70% against the wild type
Protein subunit (ZF2001)	Anhui Zhifei Longcom Biopharmaceutical, with Institute of Microbiology, Chinese Academy of Sciences	- the positive conversion rate was 96.6% in phase II clinical trial - Slightly Effective against the B.1.35 variant
